# Enhanced Rashba Effect and Optical Absorption in 2D Janus XMoYZ_2_ (X = S/Se/Te; Y = Si/Ge; Z = N/P): A First-Principles Study

**DOI:** 10.3390/nano16060358

**Published:** 2026-03-14

**Authors:** Xiaochuan Liu, Meng Li, Ningru Shang, Peng Guo, Hongyue Song, Bin Zhao, Lin Li, Jianjun Wang

**Affiliations:** Zhengzhou Key Laboratory of Low-Dimensional Quantum Materials and Devices, School of Physics and Optoelectronic Engineering, Zhongyuan University of Technology, Zhengzhou 450007, China

**Keywords:** spintronics, Rashba effect, 2D Janus XMoYZ_2_, optical absorption, first-principles

## Abstract

To overcome the physical constraints during the miniaturization of conventional semiconductor devices, spintronics is playing an increasingly prominent role. The Rashba effect, characterized by spin–momentum locking, has emerged as a promising solution to address challenges. Two-dimensional (2D) Janus transition metal dichalcogenides (TMDCs) break spatial inversion symmetry, creating favorable conditions for the Rashba effect. Based on first-principles calculations, 2D Janus materials XMoYZ_2_ (X = S/Se/Te; Y = Si/Ge; Z = N/P) were investigated, with strain, external electric field and charge doping employed to modulate the Rashba effect. The strain results reveal that the Rashba constants of XMoYZ_2_ increase significantly with compressive strain. Specifically, after applying uniaxial strain, the Rashba constant of TeMoSiP_2_ is enhanced to ~2.2 times its initial value. Compressive strain reduces atomic spacing, enhances orbital overlap, and increases spin–orbit coupling (SOC) strength. All the TeMoYZ_2_ materials exhibit significant anisotropy under uniaxial strain, which is favorable for spin-oriented transport. SeMoGeP_2_ shows an almost linear Rashba constant–electric field correlation, while TeMoGeP_2_ and TeMoSiP_2_ show non-monotonic variation. The Rashba constant of TeMoSiP_2_ can be enhanced to ~2.7 times its intrinsic value under either positive or negative applied electric fields. Charge doping induces negligible changes in the SOC effect. Finally, the optical absorption properties of TeMoGeP_2_, TeMoSiN_2_, and TeMoSiP_2_ were investigated. This study clarifies the mechanism underlying the enhancement of Rashba constants in XMoYZ_2_ materials, enriching the research landscape of spintronics.

## 1. Introduction

As Moore’s Law approaches physical limit, researchers have leveraged spin transport to mitigate quantum tunneling induced by charge-based transport. With the advancement of spintronics, the Rashba effect [1,2,3,4,5,6], arising from the effective magnetic field generated by moving electrons, has emerged as a research hotspot. It establishes a correlation between electron momentum and spin orientation via a specific energy term [7,8]. The Rashba effect can be manipulated during spin transport processes, achieving spin filtering through valley polarization [9,10]. A strong Rashba effect implies that a weak external field can achieve modulation of spin directions, reducing energy consumption [11]. Based with spin–momentum locking, the Rashba effect enables novel architectures to overcome bottlenecks, paving the way for high-performance devices [12].

Two-dimensional materials have become an ideal platform for the Rashba effect. However, as a zero-bandgap semiconductor, graphene is unfavorable for discrete information storage, and the stability of black phosphorus and the carrier mobility of traditional 2D materials pose challenges; although strong Rashba effects can been observed in several bulk materials, it remains unclear whether such effects can persist at the atomic scale [13]. Recent studies have demonstrated that 2D Janus materials exhibit excellent Rashba effects and spin tunability, attributed to their spatial inversion asymmetry [14,15,16], and transition metal dichalcogenides (TMDCs) have emerged as a focus [17,18]. These materials possess suitable bandgaps and intrinsic out-of-plane electric fields induced by different vertically aligned atoms [19,20,21,22,23]. Reports have revealed that such intrinsic fields exhibit superior efficacy in enhancing the Rashba effect compared to externally electric fields.

The Rashba effect typically occurs in confined two-dimensional electron gas systems. The Bychkov–Rashba Hamiltonian can be described by the k⋅p model [24].

(1) $$H_{R}=\alpha (\sigma \times p)\cdot \overset{\wedge }{z}=\alpha (\sigma_{x}p_{y}-\sigma_{y}p_{x})$$

where α denotes the Rashba constant, $p=\left(\begin{array}{c} p_{x}\\ p_{y} \end{array}\right)$ represents the momentum operator, $\hat{z}$ is the out-of-plane unit normal vector, and the spin operator $\sigma =\left(\sigma_{x},\sigma_{y},\sigma_{z}\right)$ corresponds to the Pauli spin matrices.

The Hamiltonian of the system can be expressed by the following formula.

(2) $$H=\frac{p^{2}}{2m^{*}}+V_{0}(r)+\frac{e\hbar^{2}E_{z}}{4m^{*2}c^{2}}(\sigma_{x}p_{y}-\sigma_{y}p_{x})$$

where the first term represents the kinetic energy term ($m^{*}$: effective mass), the middle term denotes the external electric potential, the last term corresponds to the Rashba SOC Hamiltonian, and $E_{z}$ indicates the perpendicular electrostatic potential gradient.

Let

(3) $$\alpha_{R}=\frac{e\hbar^{2}E_{z}}{4m^{*2}c^{2}}$$

The eigenvalues of the Rashba effect are derived as

(4) $$E_{R_{\pm }}=\frac{\hbar^{2}k^{2}}{2m^{*}}\pm \alpha_{R}k$$

The Rashba constant $\alpha_{R}$ is given by the quotient of the difference in energy and difference in momentum from the splitting point to the VBM or the CBM

(5) $$\alpha_{R}=2\frac{E_{R}}{k_{R}}$$

In this paper, Rashba splitting occurs in the valence band; $E_{R}$ and $k_{R}$ are defined as

(6) $$E_{R}=\left|E_{\Gamma }-E_{\max}\right|$$

(7) $$k_{R}=\left|k_{\Gamma }-k_{\max}\right|$$

where $E_{\max}$ and $E_{\Gamma }$ represent the maximum energy along two directions around and at the Γ point, respectively; $k_{\max}$ and $k_{\Gamma }$ are the momentum values corresponding to $E_{\max}$ and $E_{\Gamma }$.

For uniaxial strain, the lattice constants along the *x*-axis follow

(8) $$a=a_{0}\left(1+\varepsilon_{x}\right)$$

The other lattice constants follow

(9) $$b_{x}=b_{0x}\left(1+\varepsilon_{x}\right),\;b_{y}=b_{0y}\left(1+\varepsilon_{x}\right)$$

Freitas et al. pointed out that the density of states, bandgap type and size, orbital contributions, and topological insulating properties of XBi and XBi_3_ all undergo changes when SOC was considered, demonstrating that SOC can influence the topological properties [25]. Szczęśniak proposed the concept of metal induced gap states, transforming the insulating gap into metal like through altering the electronic distribution, governing Fermi level pinning, and mediating valley–spin polarized charge injection and transport, predicting that such states could mediate valley-resolved and spin-resolved charge transport near the electrode/MX_2_ interface, thereby resulting in valley–spin filtering [10]. Compared with reports on the strong Rashba effect in BiTeI monolayers, XMoYZ_2_ exhibits significant anisotropy, which can generate spin currents in a directional manner. This is an advantage that is difficult to achieve with BiTeI. Even though $\alpha_{R}$ has not reached a record value, its anisotropy and controllability provide a new dimension for spin devices [26,27]. Wei et al. screened 26 Janus MAA’Z_x_Z’_(4−x)_ layers and found that the Rashba effect in these materials is dominated by Se/Te-p_z_ orbitals [2]. The $\alpha_{R}$ reached 0.89 eV·Å, revealing that the intensity of the Rashba effect is closely correlated with the built-in electric field and the SOC strength. Farooq et al. showed that spin–momentum locking can be switched by a gate voltage in AB’ stacked InSe [28]. Rezavand et al. investigated 15 Janus TMDC stacking structures and found that Rashba splitting only exists in adjacent stacks with different chalcogen atoms; this effect is the most pronounced in AA-XY and A’B-XY stacking configurations, and can be significantly enhanced by an out-of-plane electric field or appropriate in-plane strain [29,30]. Hu et al. applied a high electric field of 0.5 V/Å to WSeTe, increasing its Rashba constant by 0.31 eV·Å, which confirmed the feasibility of two-dimensional materials under high electric fields [31]. These studies have shown that the strength of Rashba spin splitting can be significantly tuned by strain [32]. Su et al. studied the Rashba effect of SnS and SnSe monolayers, and found that applying strain along the ZZ, AC and biaxial directions could not only induce a bandgap transformation, but also effectively modulate the momentum shift and splitting strength [33]. Specifically, the $\alpha_{R}$ of the SnS monolayer reached 0.76 eV·Å under 6% ZZ tensile strain while that of SnSe reached 1.33 eV·Å under 2% biaxial tensile strain. In the experiment, Sibatov et al. proposed that XMoSiN_2_ can be achieved by substituting chalcogen elements for SiN_2_ on one side of MoSi_2_N_4_ [27], and MoSi_2_N_4_ has been successfully prepared experimentally by chemical vapor deposition (CVD) [34]. Strain can be realized through epitaxial growth on substrates with lattice or thermal expansion mismatch and direct stretching or compressing of flexible substrates [35].

The XMoYZ_2_ (X = S/Se/Te, Y = Si/Ge, Z = N/P) series is selected to investigate the structure–property relationships. Chalcogen X is used to tune spin–orbit coupling strength, group-IV Y modulates the electronic structure near the Fermi level, and pnictogen Z (N/P) provides complementary bonding and valence orbital features. This allows us to clarify their individual effects on the electronic and spin-related properties. Zamanian et al. reported the intrinsic Rashba constants of XMoYZ_2_ but the modulations for enhancing Rashba constants were not elaborated [25]. Therefore, the strain, electric field and charge doping were adopted to improve the Rashba constants.

## 2. Computational Methods

First-principles calculations were performed, with the Perdew–Burke–Ernzerhof (PBE) exchange correlation [36,37]. The Projector Augmented Wave (PAW) was adopted [38]. The cutoff energy of 500 eV was set for all calculations. The convergence criterion for electronic iterations was fixed at 10^−8^ eV while the ionic relaxation was 10^−4^ eV/Å. Gaussian broadening with a width of 0.05 eV was used. A 15 × 15 × 1 Monkhorst–Pack k-mesh was employed for structural optimization and optical absorption calculations. Too dense k-point samples would result in the same energy corresponding to different k-points; therefore, selecting 301 samples is sufficient enough to ensure the accuracy of Δk. The out-of-plane lattice constant was extended to 30 Å to ensure an approximately 15 Å vacuum layer. Bader charge analysis was incorporated to quantify the charge transfer [39]. The SOC was considered in all calculations.

Among all the modulations, the atoms’ relaxation was first calculated for all sampling points. When applying strain, we fixed the calculated lattice constants. When applying electric fields, the dipole correction was activated and its position was set at the center; the electric fields were set to gradually increase along the z axis. Valence electron numbers ± ne means that 1 e per unit cell is equivalent to about 0.0082 e per Å^2^. (For instance, taking the TeMoSiP_2_ lattice constant as an example, when doping with 0.1 e, it means that 10 unit-cells are doped with 1 e, and the charge density is $\frac{1}{3.493^{2}\times 10}\approx 0.0082\left(e/{Å}^{2}\right)$).

Phonon dispersions were calculated to evaluate the dynamical stability. The force constants were obtained via Density Functional Perturbation Theory (DFPT) [40,41]. The unit cell was expanded to 2 × 2 × 1, and a 5 × 5 × 1 k-mesh was used.

## 3. Results and Discussion

### 3.1. Structure

**Figure 1:**
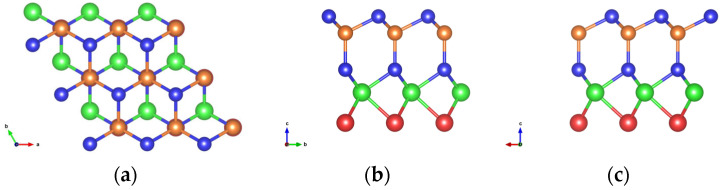
The structure of XMoYZ_2_. (**a**) Top view and (**b**), (**c**) side views; the blue, orange, green and red balls represent Z, Y, Mo, and X elements, respectively.

As illustrated in Figure 1a–c, XMoYZ_2_ structures crystallize in the hexagonal crystal system. Based on this stacking structure, twelve monolayers were constructed, namely, SeMoGeN_2_, SeMoGeP_2_, SeMoSiN_2_, SeMoSiP_2_, SMoGeN_2_, SMoGeP_2_, SMoSiN_2_, SMoSiP_2_, TeMoGeN_2_, TeMoGeP_2_, TeMoSiN_2_ and TeMoSiP_2_. The chalcogenides are located at the bottom layer, the transition metal Mo at the fourth layer, N/P at the third and first layers and Si/Ge occupy the second layer. The XMoYZ_2_ structures belong to the C_3_ Schönflies point group. Rotations around the axis passing through Mo and perpendicular to the monolayer plane (via C_3_^1^ and C_3_^2^ operations) can map the XMoYZ_2_ structures onto themselves. The Janus structures, induced by the asymmetric vertical layer distribution, break the C_3_h point group symmetry, which directly results in the absence of spatial inversion symmetry. The lattice constants and other critical data are summarized in Table 1, with reference values provided for comparison.

### 3.2. Stability

**Figure 2:**
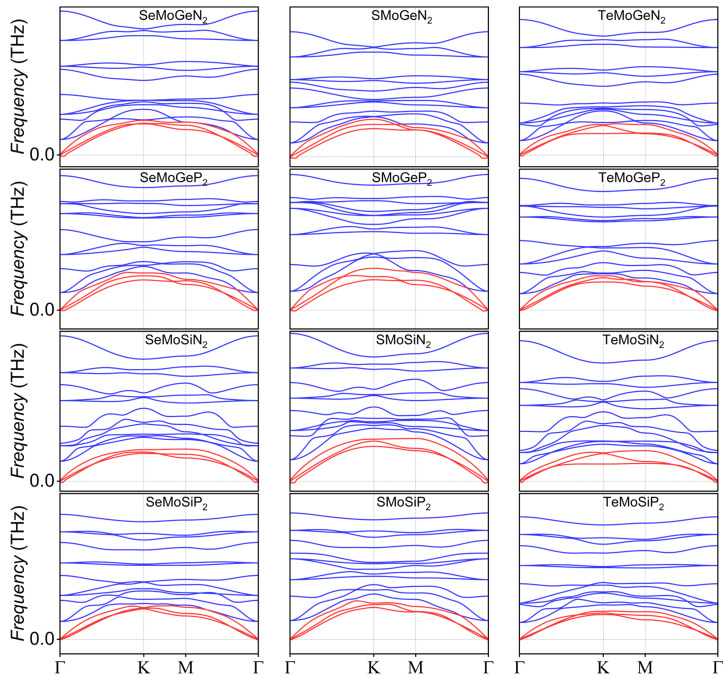
The phonon dispersion of XMoYZ_2_. The red lines represent acoustic branches, and the blue lines denote optical branches.

Figure 2 displays the phonon dispersion of XMoYZ_2_, which consists of three acoustic branches and twelve optical branches. Small imaginary frequencies are observed near the Γ point. Such weak imaginary frequencies are often not indicative of instability but rather arise from computational artifacts or finite size, which means that their frequencies are easily affected by noise in force calculations, insufficient relaxation, or inadequate sampling of the Brillouin zone, all of which can introduce small imaginary frequencies, particularly for low-energy flexural phonons [42,43].

### 3.3. Potential Energy and Charge Analysis

**Figure 3:**
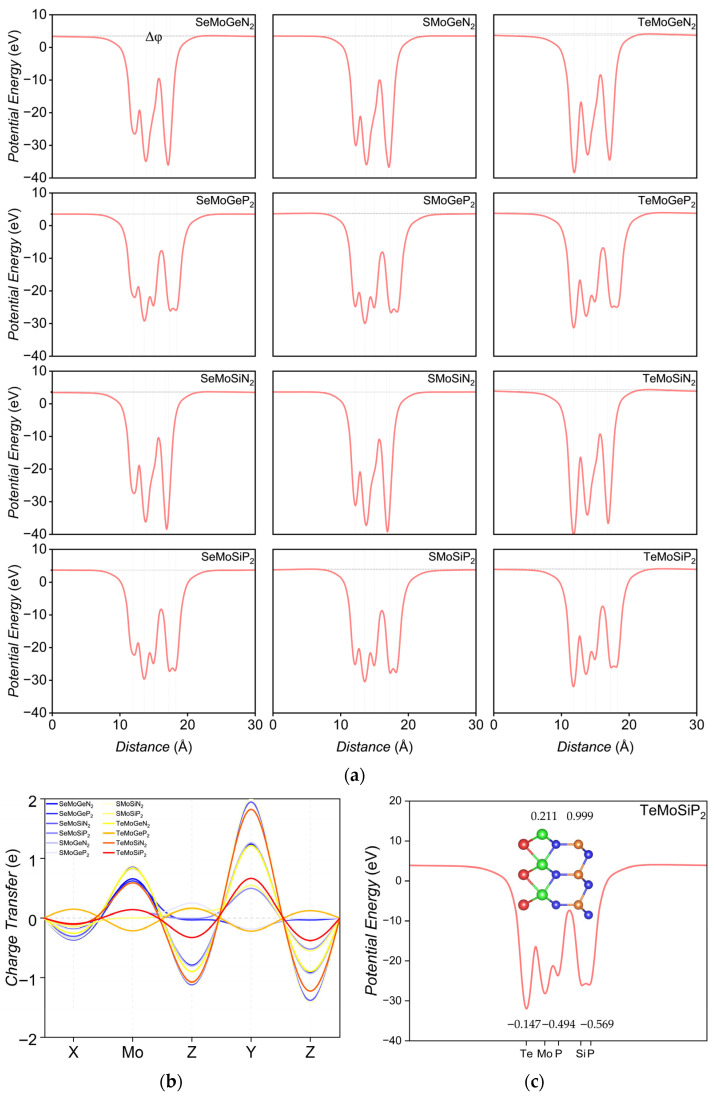
(**a**) Potential energy profile of XMoYZ_2_ as a function of distance, with horizontal lines representing the potential difference Δϕ. (**b**) Charge transfer characteristics of XMoYZ_2_; positive values denote charge depletion, while negative values signify charge accumulation. (**c**) Potential energy profile and charge transfer of TeMoSiP_2_.

Based on Figure 3a and Table 2, TeMoSiN_2_ exhibits the largest potential difference, which corresponds to the other studies [22]. The high electronegativity of N atoms endows them with a stronger electron-attracting capability than P atoms, leading to a larger electronegativity difference between N and Y/Mo atoms. This electronegativity discrepancy results in remarkable charge transfer in XMoYN_2_ materials. For instance, for TeMoSiN_2_, the charge transfer of the two N layers reaches −1.613 e and −1.842 e, respectively, which contributes to the stronger vertical intrinsic electric field. This electric field may exhibit high coupling efficiency with the Mo-d_z_^2^ orbital, thereby enhancing the Rashba effect. In contrast, the electronegativity difference between P atoms and other constituent elements is relatively small, which consequently weakens the Rashba effect. The electronegativity of Te and Mo atoms is nearly equal, which makes the electron cloud of the Mo-d_z_^2^ orbital less likely to be attracted by the Te layer; instead, it tends to hybridize with the Z_2_-p_z_ and Y-p_z_ orbitals. This result may enhance the SOC strength, rendering the Rashba constants of TeMoYZ_2_ materials more sensitive to the modulation. In contrast, the electronegativity difference between S/Se and Mo atoms may induce a shift in the Mo-d_z_^2^ orbital electron cloud toward the X layer, which may impair hybridization efficiency with the p_z_ orbitals of the top layers. This further leads to weakened SOC, resulting in the Rashba effect and modulation potential of SMoYZ_2_ or SeMoYZ_2_ being inferior to those of TeMoYZ_2_ counterparts.

Figure 3b,c reveal the intrinsic electric field inside the monolayers. Specifically, for TeMoSiP_2_, the Te layer loses a net charge of 0.147 e, while the Mo layer and its adjacent P layer accumulate and lose charges of −0.211 e and 0.494 e, respectively. The Si layer gains a net charge of 0.999 e, and the outermost P layer loses a net charge of 0.569 e. Notably, the charge transfer of SMoGeP_2_ and TeMoGeP_2_ is opposite to that of most other monolayers. In case of TeMoGeP_2_, for example, the Te layer loses a net charge of 0.221 e, while the Mo layer gains a net charge of 0.321 e; the P_1_, Ge, and P_2_ layers undergo charge changes of 0.248 e, 0.330 e, and 0.183 e, respectively. The charge accumulation and depletion for each layer are summarized in Table 2.

### 3.4. Spin Texture

**Figure 4:**
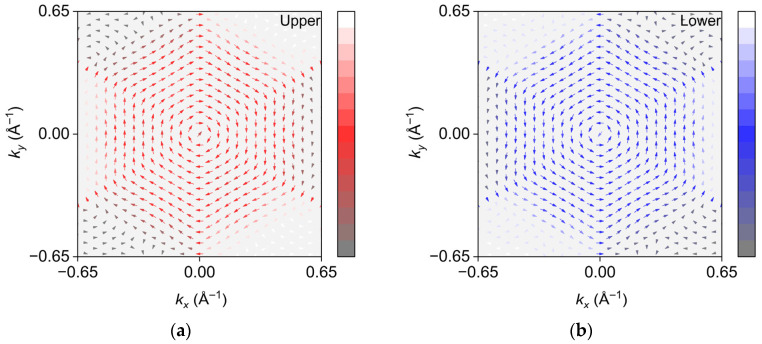
Spin textures of TeMoSiP_2_; the arrow directions indicate the spin directions. Red represents in-plane spin-up, blue represents in-plane spin-down, and the arrows lengths and depths represent the in-plane spin component magnitude. White represents out-of-plane spin-up, and dark gray represents out-of-plane spin-down. (**a**) Upper energy surface and (**b**) lower energy surface.

Taking TeMoSiP_2_ as an example, in Figure 4a, the degeneracy is lifted in the two energy surfaces where Rashba splitting occurs. Around the Γ point, the spin orientation is clockwise on the upper energy surface but counterclockwise on the lower one, displaying opposite spin chirality. Away from the Γ point, the spin direction begins to develop outward and also exhibits the opposite spin chirality, which confirms that it is Rashba spin splitting.

### 3.5. Electronic Structure

**Figure 5:**
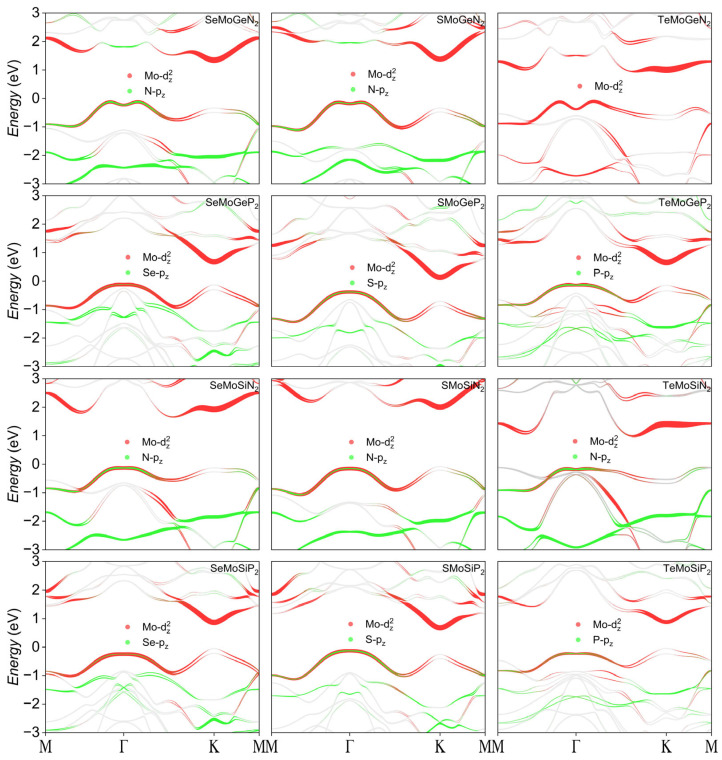
Electronic structures of XMoYZ_2_ monolayers with SOC considered; orbitals that make significant contributions to Rashba splitting are marked.

As illustrated in Figure 5, when SOC is considered, the degeneracy of the electronic structures vanishes. Among the XMoYZ_2_ monolayers, SeMoSiP_2_, SMoSiN_2_, and TeMoSiP_2_ exhibit direct bandgaps at the K point. Except for XMoGeN_2_, which shows Mexican hat-like splitting around the Γ point, all other monolayers display typical Rashba splitting. Specifically, the Rashba splitting of SeMoGeP_2_, SeMoSiN_2_, SMoGeP_2_, SMoSiP_2_, and TeMoGeP_2_ is observed at the VBM. Relevant data are summarized in Table 3. When electrons or holes accumulate at the CBM and the VBM exhibits Rashba splitting, this can provide favorable conditions for spin polarization, which shows great promise in spintronic devices. The Mo-d_z_^2^ orbitals make significant contributions to Rashba splitting at the Γ point in almost all monolayers. A plausible reason is that the Mo-d_z_^2^ orbitals extend along the z-direction, while the electron clouds of elements such as N, P, and Se are also asymmetrically distributed along the z-direction, generating internal electric fields. The Mo-d_z_^2^ orbitals couple with these electric fields, enabling the SOC effect to be maximized and thus dominating spin splitting. In some monolayers, the Mo-d_z_^2^ orbitals match the p_z_ orbitals, forming hybrid orbitals; consequently, Rashba splitting is mainly composed of the hybridization between Mo-d_z_^2^ and p_z_ orbitals.

### 3.6. Rashba Modulation

In the subsequent modulations, all structures satisfied the force convergence criteria and were confirmed to be stable.

#### 3.6.1. Strain

**Figure 6:**
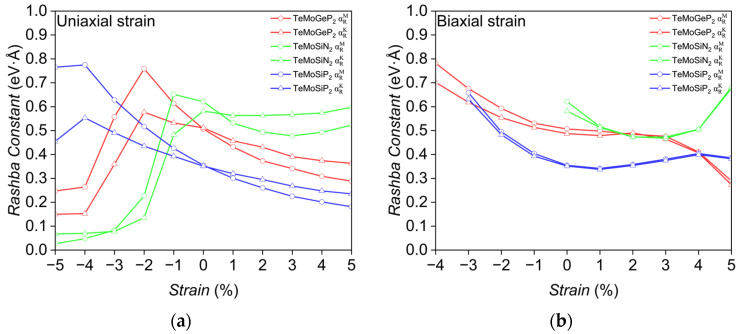
Variation in Rashba constants for TeMoYZ_2_ under different strain types: (**a**) uniaxial strain and (**b**) biaxial strain. The red, green, and blue lines represent TeMoGeP_2_, TeMoSiN_2_, and TeMoSiP_2_, respectively. Circular lines correspond to the Γ→M direction, while triangular lines correspond to the Γ→K direction.

**Figure 7:**
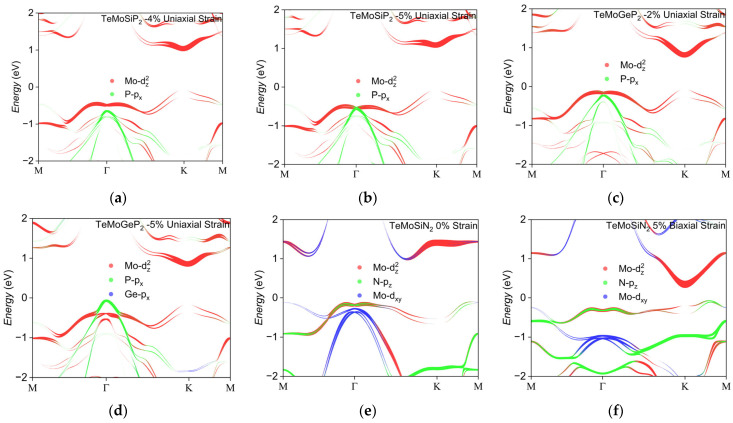
The electronic structures trend of TeMoSiP_2_, TeMoGeP_2_ and TeMoSiN_2_ under different kinds and degrees of strain. (**a**,**b**) TeMoSiP_2_ under −4% and −5% uniaxial strain, (**c**,**d**) TeMoGeP_2_ under −2% and −5% uniaxial strain and (**e**,**f**) TeMoSiN_2_ under 0% and 5% biaxial strain.

TeMoYZ_2_ was selected as the research object due to its excellent performance. Combining Figure 5 and Figure 6a, under uniaxial strain, the Rashba constant of TeMoSiP_2_ increases rapidly with the enhancement of compressive strain, with an intrinsic Rashba constant of 0.351 eV·Å. At this stage, the Mo-d_z_^2^ orbital is the main contributor, accompanied by the participation of a small amount of P-p_z_ and Si-p_z_ orbitals. Subsequently, upon applying −4% compressive strain, the Rashba constants along the Γ→M and Γ→K directions increase to 0.775 eV·Å and 0.553 eV·Å, respectively, which are 2.21 and 1.58 times the intrinsic value; the proportion of Mo-d_z_^2^ is 39.6% and that of P-p_x_ is 0.6%. From Table 4, the wave vector k where the peaks are located is shifting away from Γ, but ΔE increases faster than Δk. However, with the increase in compression, the Rashba constant shows a downward trend, which is attributed to the upward shift in the deeper energy levels in the valence band dominated by P-px orbitals; the proportion of Mo-d_z_^2^ is 17.9% and that of P-p_x_ is 17.9%. As illustrated in Figure 7a,b, these energy levels subsequently couple with Mo-d_z_^2^ orbitals and engage in Rashba splitting.

Similarly, TeMoGeP_2_ also has an intrinsic Rashba constant of approximately 0.511 eV·Å, which is slightly higher than that of TeMoSiP_2_. For the initial Rashba effect, the Mo-d_z_^2^ orbital is the main contributor, followed by the P-p_z_ orbital. As shown in Figure 6a, under a compressive strain of −2%, the Rashba constants reach their maximum values: 0.759 eV·Å along the Γ→M direction and 0.577 eV·Å along the Γ→K direction. This is because ΔE increases continuously while Δk decreases compared to that under −1% or 0% strain, leading to an increase in the Rashba constant. However, after exceeding this threshold strain, a downward trend also occurs. As shown in Figure 7c,d, when the compressive strain increases from −2% to −5%, the Rashba splitting region originally dominated by the Mo-d_z_^2^ orbital is completely replaced by the deeper-lying P-p_x_ orbitals; the proportions of Mo-d_z_^2^ and P-p_x_ change from 33% and 4.8% to 0.2% and 36.6%, respectively, showing a prominent profile, resulting in almost no typical Rashba effect near the Fermi level, and the ratio of ΔE to Δk becomes extremely small.

Among the three monolayers, TeMoSiN_2_ exhibits the largest intrinsic Rashba constant, with values along the two directions reaching 0.621 eV·Å and 0.582 eV·Å, respectively, as shown in Figure 6a. With the increase in tensile strain, the Rashba constant remains stable along the Γ→K direction, indicating that it has good robustness against uniaxial stretching along this direction.

As shown in Figure 6b, under biaxial strain, a compressive strain of −3% can enhance the Rashba constants of TeMoSiP_2_ to approximately 0.659 eV·Å and 0.632 eV·Å along the two directions, which are about 1.88 and 1.81 times the intrinsic values, respectively. The Rashba constant of TeMoGeP_2_ increases continuously with the enhancement of compressive strain; under a compressive strain of −4%, the Rashba constants are increased to 0.782 eV·Å and 0.702 eV·Å. However, both TeMoGeP_2_ and TeMoSiN_2_ show a trend of first decreasing and then increasing under tensile strain. For TeMoSiN_2_, the splitting originally dominated by the Mo-d_z_^2^ and N-p_z_ orbitals gradually transforms into a Mexican hat-type splitting, as shown in Figure 7e,f; the proportion of Mo-d_z_^2^ is 41.1% and that of P-p_x_ is 19.7%.

Most monolayers exhibit more significant anisotropy under uniaxial strain. For example, under a −4% uniaxial compressive strain, the Rashba constants of TeMoSiP_2_ along the Γ→M and Γ→K directions differ by 0.222 eV·Å. When TeMoGeP_2_ is subjected to a −2% biaxial compressive strain, this difference is only 0.039 eV·Å, but it reaches 0.182 eV·Å under uniaxial strain.

#### 3.6.2. Electric Field

**Figure 8:**
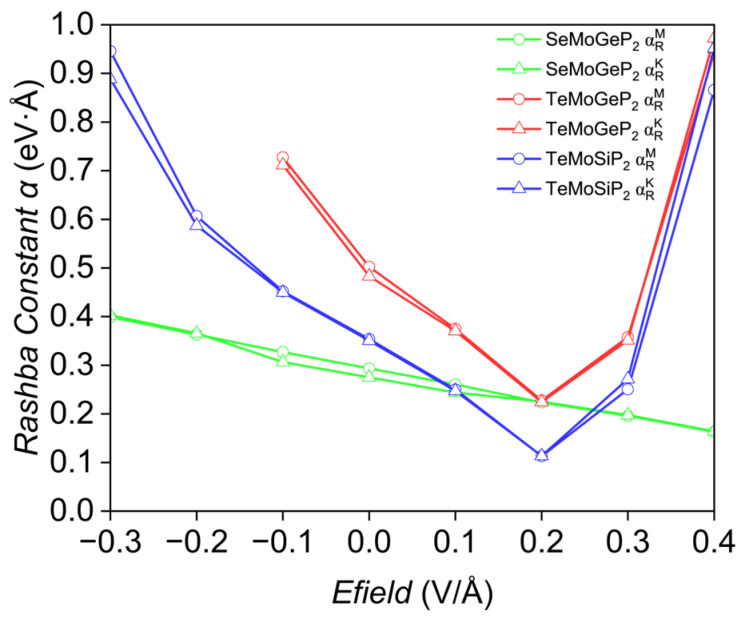
The variation in the Rashba constants with the electric field, the green, red, and blue lines represent SeMoGeP_2_, TeMoGeP_2_ and TeMoSiP_2_, respectively.

As illustrated in Figure 8, the Rashba constants of SeMoGeP_2_ exhibit an almost linear correlation with the applied electric field, where a negative electric field can enhance the Rashba constants to a certain degree. However, such a typical linear relationship is not universal among XMoYZ_2_ compounds.

**Figure 9:**
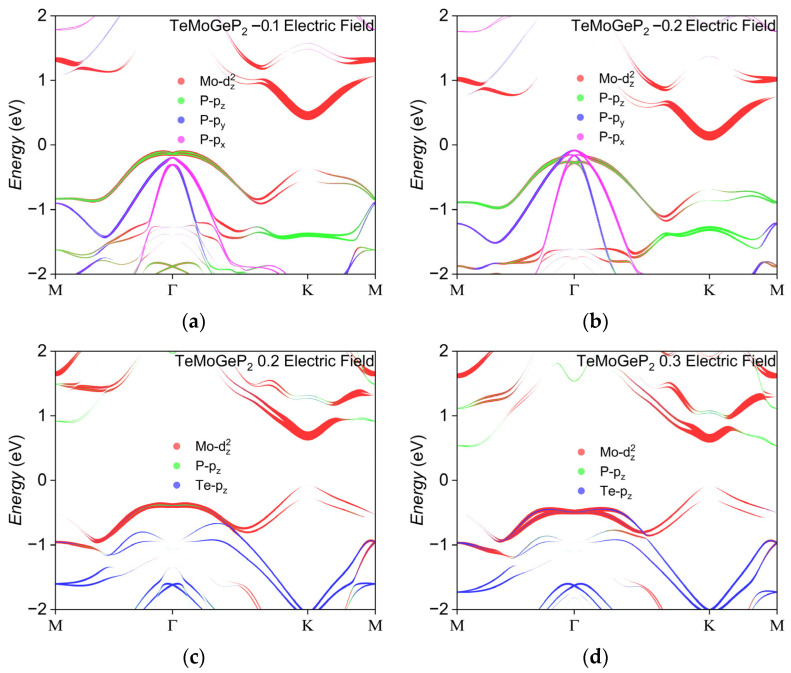
The electronic structure changes of TeMoGeP_2_ under different electric fields: (**a**) −0.1 V/Å, (**b**) −0.2 V/Å, (**c**) 0.2 V/Å and (**d**) 0.3 V/Å.

Figure 9 depicts the electronic structure of TeMoGeP_2_ under different electric fields. When the electric field reaches −0.1 V/Å, the Rashba constants increase to 0.727 eV·Å and 0.712 eV·Å, respectively. Beyond this threshold, the P-p_x_ and P-p_y_ orbitals which do not exhibit the Rashba effect take the place of the original Mo-d_z_^2^ and P-p_z_ orbital; the proportions of Mo-d_z_^2^ and P-p_z_ change from 31.9% and 15.4% to 0% and 0%, respectively, but P-p_x_ and P-p_y_ emerge as 17.3% and 17.4%. A positive electric field induces a non-monotonic variation in the Rashba constants, characterized by an initial decrease followed by an increase. As shown in Table 5, in the electric field range of 0 V/Å to 0.2 V/Å, the Δk values along the two directions remain nearly unchanged, while ΔE decreases significantly, resulting in a reduction in the Rashba constants. It can be seen from Figure 9c,d that from 0.2 V/Å to 0.3 V/Å, around the Rashba splitting point dominated by the Mo-d_z_^2^ orbital, the proportion of Mo-d_z_^2^, P-p_z_ and Te-p_z_ change from 46%, 6.9% and 2.6% to 46%, 4.1% and 5.5%; the growth rate of ΔE exceeds that of Δk, leading to an increase in the Rashba constants. Specifically, under an electric field of 0.4 V/Å, the Rashba constant reaches approximately 0.95 eV·Å.

TeMoSiP_2_ follows a similar variation trend to TeMoGeP_2_: under an electric field of −0.3 V/Å, Δk decreases while ΔE increases, ultimately leading to Rashba constants along the Γ→K and Γ→M directions of 0.889 eV·Å and 0.946 eV·Å, respectively, 2.53 and 2.70 times the intrinsic values. When the electric field is 0.4 V/Å, the Rashba constants of TeMoSiP_2_ also reach around 0.9 eV·Å. This represents a substantial enhancement in the Rashba constants of two-dimensional materials.

#### 3.6.3. Charge Doping

**Figure 10:**
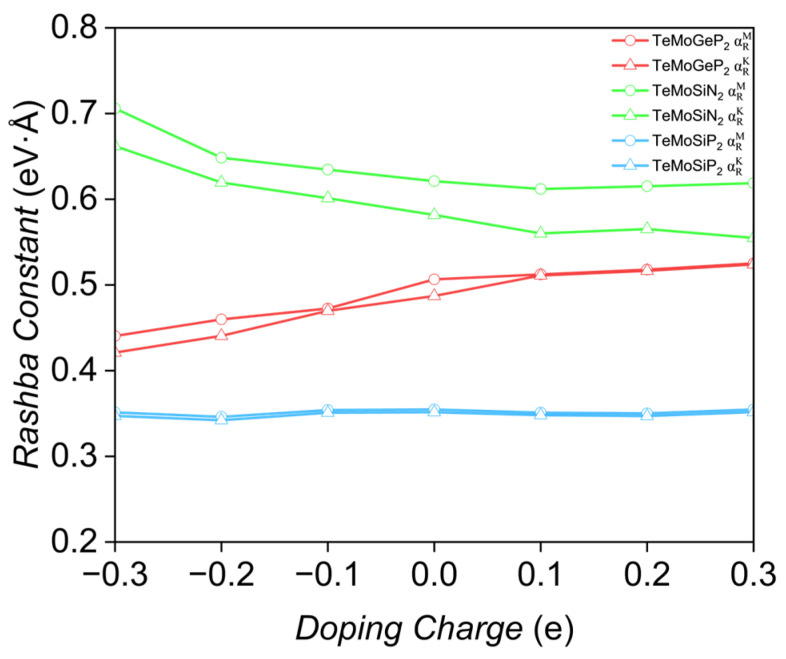
The changes in Rashba constants under charge doping, with the red, green, and blue lines representing TeMoGeP_2_, TeMoSiN_2_ and TeMoSiP_2_, respectively.

Figure 10 illustrates the variations in Rashba constants of the three monolayers under p-doping and n-doping. It is evident that all three monolayers maintain high structural stability during the doping process. For TeMoGeP_2_, the Rashba constants exhibit a slight increase under n-doping: when the doping level reaches 0.3 e (~0.028 e/Å^2^), the Rashba constants increase by 0.017 eV·Å and 0.037 eV·Å, respectively. In contrast, under p-doping (doping level of −0.3 e), the Rashba constants are 0.422 eV·Å and 0.440 eV·Å, corresponding to a decrease of only approximately 0.066 eV·Å. For TeMoSiN_2_, p-doping can moderately enhance the Rashba constants; specifically, at a doping level of −0.3 e, the Rashba constants along the two directions increase by 0.085 eV·Å and 0.08 eV·Å, respectively. TeMoSiP_2_ demonstrates high robustness against charge doping.

The Fermi level shifts slightly toward the conduction band after n-doping; conversely, p-doping leads to the Fermi level exhibiting a significant shift toward the valence band. The charge doping adjusts the Fermi level by populating or depopulating the electrons near the band edges, but hardly modifies the electronic structures and SOC strength.

We speculate that in XMoYZ_2_, varying the electron count via charge doping only shifts the Fermi energy significantly, while the Rashba constants remain nearly unchanged and are barely affected by such a moderate charge modulation.

Based on the results, we make the following speculations: the essence of strain is the adjustment of atomic spacing and bond angles; lattice distortion alters the wave function overlap integral of the key orbitals of Rashba splitting; the greater the wave function overlap integral, the higher the probability of electron transitions between different orbitals, the stronger the hybridization, and the higher the coupling efficiency of SOC. For an external electric field, the Coulomb force will change the distribution of the electron cloud, regulating the strength of the vertical intrinsic electric field and the orbital hybridization direction; this belongs to the electron state rearrangement dominated by the Coulomb force. For some XMoYZ_2_, the modulation has non-monotonicity, related to the direction and intensity of the electric field. The modulation amplitude for TeMoYZ_2_ is much greater than that for SMoYZ_2_ or SeMoYZ_2_. For charge doping, we speculate that only changing the charge density without charge transfer proportion between layers will not change the wave function shape of the orbitals; the overlap integral of Mo-d_z_^2^ and Y/Z-p_z_ orbitals will not change, and the SOC strength remains stable; the Janus structure is not destroyed, the spatial inversion asymmetry remains unchanged, and the intensity of the intrinsic electric field also remains stable.

### 3.7. Optical Properties

**Figure 11:**
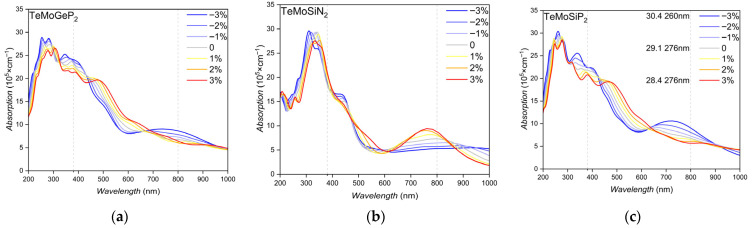
The optical absorption spectra of TeMoGeP_2_, TeMoSiN_2_ and TeMoSiP_2_ under different levels of biaxial strain. (**a**) TeMoGeP_2_; (**b**) TeMoSiN_2_; (**c**) TeMoSiP_2_.

Figure 11 reveals distinct optical absorption responses of TeMoGeP_2_, TeMoSiN_2_, and TeMoSiP_2_, particularly in the visible light and absorption peak regimes. All three monolayers generally exhibit increasingly stronger optical absorption from red light to violet light (about 380 nm−800 nm), with absorption values rapidly rising from 5–10 × 10^5^ to 15–25 × 10^5^ cm^−1^. The responses of optical absorption to compressive and tensile strains exhibit multiple reversals, providing flexibility for strain modulated optical absorption and indicating optical absorption for visible light applications. Meanwhile, their primary absorption peaks occur between 200 nm and 400 nm, where near-ultraviolet waves are located, reaching maximum intensities of 25–30 × 10^5^ cm^−1^ before gradually declining with increasing energy.

Compressive strain induces two key effects on the absorption peaks. Firstly, it enhances the peaks’ intensity; the blue curves consistently show higher absorption magnitudes than the red curves. Secondly, the absorption peaks shift toward lower wavelength compared to unstrained or tensile strain, so compressive strain may cause blue shifts. This may arise from strain-induced modifications to the electronic structures, where compressive strain may increase the bandgap energy, shifting optical transitions to shorter wavelengths. These findings demonstrate that compressive strain is an effective tool for tuning both the strength and spectral position of absorption peaks in these monolayers.

## 4. Conclusions

In summary, we performed investigations on the stability, potential, charge transfer, and SOC-considered electronic structure of XMoYZ_2_ via first-principles calculations. We focused on the modulation of Rashba constants in XMoYZ_2_ monolayers through strain, external electric fields, and charge doping. The results indicate that TeMoYZ_2_ exhibits a superior intrinsic Rashba effect, which becomes more prominent under strain modulation. Specifically, a −2% uniaxial strain can increase the Rashba constant of TeMoGeP_2_ from 0.507 eV·Å to 0.759 eV·Å, and a −4% uniaxial strain can increase the TeMoSiP_2_ Rashba constant from 0.355 eV·Å to 0.775 eV·Å. Moreover, −0.3 V/Å and 0.4 V/Å external electric fields can raise the Rashba constants of TeMoSiP_2_ from about 0.35 eV·Å to around/1 eV·Å. TeMoYZ_2_ demonstrates robustness against charge doping and can slightly enhance the Rashba constants of TeMoGeP_2_ and TeMoSiN_2_. Finally, the optical absorption efficiency of TeMoYZ_2_ was tested and it was found that TeMoSiN_2_ has strong optical selectivity. In the ultraviolet region, compressive strain can induce a blue shift in the absorption peak and enhance absorption intensity. Through this study, we achieved a more than twofold enhancement in the Rashba constants of several XMoYZ_2_ materials, which holds substantial promise for applications in electronic devices. The synthesis of XMoYZ_2_ monolayers is expected via mature chemical vapor deposition, and precise crystallinity is crucial for the predicted topological properties. These findings not only enrich the theoretical basis for regulating the Rashba effect in 2D Janus materials but also provide guidance for the design and development of high-performance spintronic and optoelectronic devices.

## Figures and Tables

**Table 1:** The lattice constants and bond lengths of XMoYZ_2_; Z_1_ and Z_2_ represent the middle layers and the outside layers, respectively.

	α(Å), a = b	Ref. [22]	l_X-Mo_ (Å)	l_Mo-Z1_ (Å)	l_Z1-Y_ (Å)	l_Y-Z2_ (Å)
SeMoGeN_2_	3.103	3.100	2.511	2.137	1.882	1.881
SeMoGeP_2_	3.478	3.471	2.585	2.449	2.325	2.300
SeMoSiN_2_	3.013	3.009	2.499	2.115	1.752	1.802
SeMoSiP_2_	3.428	3.421	2.574	2.439	2.239	2.236
SMoGeN_2_	3.067	3.067	2.390	2.132	1.878	1.867
SMoGeP_2_	3.436	3.434	2.473	2.444	2.324	2.288
SMoSiN_2_	2.980	2.980	2.374	2.109	1.751	1.786
SMoSiP_2_	3.386	3.385	2.461	2.434	2.239	2.223
TeMoGeN_2_	3.169	3.169	2.696	2.150	1.889	1.908
TeMoGeP_2_	3.545	3.542	2.750	2.460	2.328	2.320
TeMoSiN_2_	3.077	3.077	2.690	2.130	1.755	1.833
TeMoSiP_2_	3.493	3.493	2.743	2.448	2.240	2.256

**Table 2:** The potential difference Δϕ, charge accumulation and depletion per layer of XMoYZ_2_.

	Δφ	Se/S/Te	Mo	N/P	Ge/Si	N/P
SeMoGeN_2_	0.232	−0.281	0.988	−1.191	1.861	−1.377
SeMoGeP_2_	0.018	−0.556	0.934	−0.048	−0.279	−0.050
SeMoSiN_2_	0.194	−0.464	1.299	−1.680	2.918	−2.073
SeMoSiP_2_	0	−0.161	0.209	−0.012	0.748	−0.783
SMoGeN_2_	0.027	−0.522	1.244	−1.215	1.893	−1.400
SMoGeP_2_	−0.156	−0.292	0.200	0.380	−0.279	−0.008
SMoSiN_2_	0	−0.537	1.317	−1.647	3.000	−2.133
SMoSiP_2_	−0.224	−0.225	0.001	0.233	0.822	−0.831
TeMoGeN_2_	0.460	−0.385	1.254	−1.347	1.825	−1.347
TeMoGeP_2_	0.176	0.221	−0.321	0.248	−0.330	0.183
TeMoSiN_2_	0.492	−0.167	0.885	−1.613	2.737	−1.842
TeMoSiP_2_	0.182	−0.147	0.211	−0.494	0.999	−0.569

**Table 3:** Bandgap types, locations of Rashba splitting and bandgaps with SOC (E_g_).

	VBM	CBM	Type	Location	E_g_ (PBE + SOC)
SeMoGeN_2_	Γ	K	I		1.448
SeMoGeP_2_	Γ	K	I	VBM	0.791
SeMoSiN_2_	Γ	Γ→K	I	VBM	1.774
SeMoSiP_2_	K	K	D		0.912
SMoGeN_2_	Γ	K	I		1.512
SMoGeP_2_	Γ	K	I	VBM	0.510
SMoSiN_2_	K	K	D		2.081
SMoSiP_2_	Γ	K	I	VBM	0.803
TeMoGeN_2_	Γ	K	I		1.022
TeMoGeP_2_	Γ	K	I	VBM	0.761
TeMoSiN_2_	M	Γ→K	I		1.077
TeMoSiP_2_	K	K	D		0.914

**Table 4:** The variation in ΔE and Δk with the two kinds of strains of TeMoYZ_2_.

		Uniaxial Strain (%)				Biaxial Strain (%)			
		ΔE_Γ→M_	Δk_Γ→M_	ΔE_Γ→K_	Δk_Γ→K_	ΔE_Γ→M_	Δk_Γ→M_	ΔE_Γ→K_	Δk_Γ→K_
TeMoGeP_2_	−5	0.00044	0.00355	0.00030	0.00399				
	−4	0.00093	0.00704	0.00061	0.00796	0.06253	0.15989	0.07630	0.21745
	−3	0.00487	0.01746	0.00285	0.01587	0.04990	0.14769	0.05651	0.18272
	−2	0.03415	0.09004	0.02741	0.09502	0.03818	0.12878	0.04120	0.14871
	−1	0.03054	0.09967	0.02836	0.10662	0.03016	0.11370	0.03165	0.12333
	0	0.02505	0.09892	0.02719	0.10634	0.02505	0.09892	0.02590	0.10634
	1	0.02049	0.09500	0.02336	0.10215	0.02185	0.08781	0.02241	0.09359
	2	0.01694	0.09075	0.02108	0.09799	0.01944	0.08026	0.01987	0.08109
	3	0.01424	0.08342	0.01910	0.09776	0.01656	0.06955	0.01688	0.07265
	4	0.01228	0.07952	0.01751	0.09363	0.01212	0.05903	0.01232	0.06060
	5	0.01091	0.07567	0.01625	0.08953	0.00658	0.04548	0.00667	0.04877
TeMoSiN_2_	−5	0.00005	0.00408	0.00031	0.00920				
	−4	0.00010	0.00405	0.00032	0.00917				
	−3	0.00050	0.01206	0.00054	0.01372				
	−2	0.00544	0.04788	0.00278	0.04105				
	−1	0.04255	0.13066	0.04613	0.19107				
	0	0.04271	0.13754	0.04883	0.16790	0.04271	0.13754	0.04883	0.16790
	1	0.03727	0.14043	0.04587	0.16295	0.03716	0.14397	0.04032	0.15725
	2	0.03441	0.13940	0.04452	0.15804	0.03470	0.14640	0.03683	0.15571
	3	0.03398	0.14224	0.04470	0.15768	0.03571	0.15261	0.03751	0.15860
	4	0.03576	0.14505	0.04640	0.16181	0.04005	0.15871	0.04190	0.16580
	5	0.03962	0.15162	0.04958	0.16593	0.05822	0.17216	0.06041	0.17719
TeMoSiP_2_	−5	0.06197	0.16197	0.04332	0.19000				
	−4	0.06223	0.16067	0.04805	0.17400				
	−3	0.04784	0.15232	0.03856	0.15700	0.05053	0.15346	0.05860	0.18545
	−2	0.03634	0.14060	0.03145	0.14500	0.03582	0.14483	0.03938	0.16315
	−1	0.02745	0.12906	0.02596	0.13225	0.02611	0.12938	0.02777	0.14132
	0	0.02087	0.11770	0.02178	0.12392	0.02087	0.11770	0.02178	0.12392
	1	0.01600	0.10652	0.01848	0.11564	0.01874	0.10968	0.01938	0.11478
	2	0.01243	0.09551	0.01585	0.10740	0.01885	0.10521	0.01943	0.10973
	3	0.00990	0.08805	0.01382	0.10318	0.02045	0.10756	0.02110	0.11254
	4	0.00813	0.08069	0.01226	0.09899	0.02219	0.10984	0.02301	0.11531
	5	0.00697	0.07679	0.01114	0.09482	0.02226	0.11539	0.02326	0.12182

**Table 5:** The variation trends of ΔE and Δk in the electric field of SeMoGeP_2_, TeMoGeP_2_ and TeMoSiP_2_.

Electric Field (V/Å)					
		ΔE_Γ→M_	Δk_Γ→M_	ΔE_Γ→K_	Δk_Γ→K_
SeMoGeP_2_	−0.3	0.01040	0.05215	0.01051	0.05219
	−0.2	0.00946	0.05215	0.00955	0.05219
	−0.1	0.00853	0.05215	0.00861	0.05621
	0	0.00833	0.05215	0.00841	0.05621
	0.1	0.00680	0.05215	0.00686	0.05621
	0.2	0.00583	0.05215	0.00588	0.05219
	0.3	0.00511	0.05215	0.00515	0.05219
	0.4	0.00425	0.05215	0.00428	0.05219
TeMoGeP_2_	−0.1	0.02853	0.07845	0.02943	0.08271
	0	0.02485	0.09892	0.02568	0.10634
	0.1	0.01980	0.10574	0.02043	0.11028
	0.2	0.01202	0.10574	0.01237	0.11028
	0.3	0.03903	0.21830	0.04359	0.24814
	0.4	0.13178	0.27970	0.16082	0.33085
TeMoSiP_2_	−0.3	0.04419	0.09346	0.04618	0.10393
	−0.2	0.03358	0.11077	0.03520	0.11992
	−0.1	0.02660	0.11770	0.02787	0.12392
	0	0.02082	0.11770	0.02173	0.12392
	0.1	0.01385	0.11077	0.01434	0.11593
	0.2	0.00509	0.09000	0.00521	0.09194
	0.3	0.02948	0.23540	0.04291	0.31579
	0.4	0.12588	0.29079	0.17120	0.35976

## Data Availability

Data are contained within the article.
